# Validation and multi-site deployment of a lyophilized qRT-PCR reagent for the molecular diagnosis of avian influenza and rabies in Sub-Saharan African regions

**DOI:** 10.1128/jcm.00080-25

**Published:** 2025-07-01

**Authors:** Petra Drzewnioková, Irene Brian, Marzia Mancin, Andrea Fortin, Morgane Gourlaouen, Angélique Angot, Mamadou Niang, Isaac Dah, Kouramoudou Berete, Adama Diakite, Fatou Tall Lo, Clement Meseko, Emilie Go-Maro, Valeria D’Amico, Viviana Valastro, Baba Soumare, Paola De Benedictis, Isabella Monne, Valentina Panzarin

**Affiliations:** 1 Istituto Zooprofilattico Sperimentale delle Venezie 83372, Legnaro, Padua, Italy; 2 Department of Public Health, University of Pavia 19001, Pavia, Italy; 3 Food and Agriculture Organization of the United Nations 17107, Rome, Italy; 4 Food and Agriculture Organization of the United Nations 247369, Accra, Ghana; 5 Laboratoire National Vétérinaire de Yaoundé, Yaoundé, Cameroon; 6 Laboratoire Central de Diagnostic Vétérinaire de Conakry, Conakry, Guinea; 7 Laboratoire Central Vétérinaire de Bamako, Bamako, Mali; 8 Laboratoire National d’Elevage et de Recherches Vétérinaires de Dakar, Dakar, Senegal; 9 National Veterinary Research Institute 206895, Vom, Nigeria; 10 Laboratoire Central Vétérinaire de Lomé, Lomé, Togo; University of California, Davis, Davis, California, USA

**Keywords:** lyophilized reagent, avian influenza, rabies, molecular methods, RT-PCR

## Abstract

Molecular methods are widely accepted as gold-standard techniques for the laboratory diagnosis of most human and animal pathogens. However, most molecular protocols rely on reagents that need to be transported and stored at a freezing temperature, a requirement that might affect their reliability in areas where the cold chain cannot be guaranteed. Over the years, several lyophilized molecular products have been marketed to circumvent this issue. We therefore evaluated the feasibility of replacing liquid reagents with freeze-dried formulations for the molecular diagnosis of avian influenza (AIV) and rabies (RABV) viruses, two priority zoonotic pathogens widely spread in Sub-Saharan Africa. Among six different commercial freeze-dried kits, we selected one reagent due to its easy-to-use features, single-reaction format, and preliminary performance assessment. Through a more in-depth evaluation, we determined its analytical and diagnostic performance and formulation stability and obtained results comparable to those of standard liquid master mixes. However, for the detection of divergent lyssaviruses, the lyophilized reagent’s sensitivity was affected by suboptimal complementarity between the oligonucleotides and target sequences. Finally, a multi-site evaluation in four veterinary diagnostic laboratories located in Sub-Saharan Africa demonstrated the successful deployment of AIV and RABV assays utilizing the freeze-dried reagent, which can interchangeably replace liquid qRT-PCR/RT-PCR kits. Altogether, our results indicate that the investigated lyophilized master mix represents a valid alternative to liquid reagents for the molecular diagnosis of avian influenza and rabies and has the potential for broader applications to other relevant infectious diseases upon proper validation.

**IMPORTANCE:**

Molecular diagnostic protocols rely on reagents that need to be transported and stored at freezing temperatures. Meeting this requirement can be challenging in areas where the maintenance of the cold chain is not guaranteed, such as in Sub-Saharan Africa. Our study aimed to assess the feasibility of using lyophilized reagents as a replacement for liquid reagents in the molecular diagnosis of two widespread zoonotic pathogens in Sub-Saharan Africa, namely, avian influenza and rabies. To accomplish this, we selected a commercially available lyophilized reagent based on its format and performance characteristics. We conducted a laboratory validation to assess the use of the lyophilized reagent throughout the entire diagnostic process. We also conducted a reproducibility test involving African laboratories as potential end-users. Our findings confirm that the lyophilized reagent can replace traditional liquid reagents to diagnose rabies and avian influenza and suggest its possible use for a wider range of infectious diseases after undergoing appropriate validation.

## INTRODUCTION

The strong connections between the health of humans, animals, and the ecosystems recognized by the One Health holistic approach have led to the establishment of the Global Plan of Action for One Health (GPA) shared within the quadripartite alliance (1, 2) among the Food and Agriculture Organization of the United Nations (FAO), the UN Environment Programme (UNEP), the World Health Organization (WHO), and the World Organization for Animal Health (WOAH). The GPA identifies as top priorities not only emerging zoonotic diseases with epidemic and pandemic potential but also neglected and endemic diseases of zoonotic and vector-borne origin (2). Indeed, while the disruptive impact of epidemics and pandemics caused by the emergence of zoonotic pathogens appears obvious, the indirect consequences in terms of missed opportunities for the control, prevention, and treatment of endemic and neglected diseases should not be overlooked (3–5). In this view, zoonotic influenza (including avian influenza, AI) and rabies have been long recognized as potential showcases for the One Health inter-sectorial collaboration (6–8).

AI is a highly contagious disease caused by influenza A viruses (AIV; genus *Alphainfluenzavirus,* family *Orthomyxoviridae*), which can be classified based on their levels of pathogenicity to chickens or the composition of the hemagglutinin (HA) cleavage site, resulting in designations as either low-pathogenicity avian influenza (LPAI) or high-pathogenicity avian influenza (HPAI). So far, only viruses of the H5 and the H7 subtype have proved to be able to evolve into an HPAI form, displaying a heightened ability to cause severe disease and death in infected poultry. Among the multiple subtypes of AIV reported so far in domestic birds, LPAI H9N2 and HPAI H5N1 viruses are currently the most widespread in the poultry population (9). Both subtypes can cause significant economic losses to the poultry industry, being responsible for a remarkable number of infections in mammals and for sporadic spillover events in humans (10, 11). These viral threats have a particularly devastating impact in developing countries, such as the Sub-Saharan African territories, where poultry farming is a highly developing sector counting on approximately 1.1 billion of poultries (12) and, at the same time, an important source of income for several traders and rural households (13). Indeed, HPAI H5N1 viruses of the A/Goose/Guangdong/1/1996 (Gs/GD) lineage (14) and G1-like lineage H9N2 viruses are now entrenched across the poultry population of multiple African countries. Combined with poor surveillance, limited response capacity, and deficient reporting, this complex epidemiological scenario creates the opportunity for virus evolution and paves the way for the emergence of strains with unexpected zoonotic potential (15, 16).

Rabies, an ancient although preventable disease, still kills an estimated 60,000 people every year (6) and mostly affects the poorest communities in rural Africa and Asia (17–19). Rabies can also occasionally result in livestock’s death due to spillover events, determining an income loss of 6% the 8.6 billion USD economic losses per year (20), which is expected to impact the fragile economies of endemic countries (17, 21). Despite rabies being potentially caused by all members belonging to the *Lyssavirus* genus (family *Rhabdoviridae*), rabies virus (RABV) is the most widespread and mainly responsible for human deaths worldwide. Indeed, the remaining 17 non-RABV lyssavirus species recognized to date (22) display restricted geographical and ecological niches, mostly remaining confined to their natural hosts. In Africa, six non-RABV lyssaviruses have been detected so far, namely, Duvenhage virus (DUVV) (23), Lagos Bat Virus (LBV) (24), Mokola virus (MOKV) (25), Shimoni bat virus (SHBV) (26), Ikoma virus (IKOV) (27), and the recently discovered Matlo Bat Virus (MTLBV) (28), with rare spillover infections identified in domestic animals and humans (29). Considering that infections with divergent lyssaviruses are able to cause severe clinical signs and impact on public health, international organizations recommend the use of a broad range of laboratory methods to diagnose both RABV and non-RABV lyssaviruses from animal suspect cases (30, 31). Nevertheless, rabies is maintained by a vicious circle of neglect, in which poor surveillance and reporting lead to underestimating the burden of the disease and, consequently, to making insufficient efforts for its control and prevention (6, 12, 13, 17, 21). Thus, improved rabies surveillance and diagnosis are key drivers for maintenance of human and animal health and for measuring the impact of control programs, ultimately offering reliable data to decision-makers (17, 32, 33).

In the last decades, we have witnessed the massive implementation of molecular techniques, which have greatly contributed to the success of surveillance and control programs for infectious diseases (34). Regulatory health authorities have long recognized the use of molecular testing for AIV detection and typing and largely rely on the rapidity of such methods for prompt confirmation and notification of outbreaks (35, 36). On the contrary, rabies molecular testing techniques have only recently been included in the WOAH and WHO diagnostic manuals (30, 31), albeit their suitability has been widely demonstrated by direct comparison with gold-standard methods (37, 38). So far, high-income countries have been the main beneficiaries of such fast and accurate molecular testing methods (17, 21, 39) as their settings can guarantee cold-chain maintenance for proper transportation and storage of reagents and samples (40). Unfortunately, this basic requirement is a struggle in low- and middle-income countries of tropical and subtropical areas, whose operability is often prevented by inadequate investments and unfavorable climate conditions (41) despite the technical skills achieved by the laboratory personnel through application of consolidated cooperation programs (42, 43). Actually, we are aware of the difficulty in maintaining the recommended temperature for storing samples after delivery to diagnostic laboratories, and we well understand that such inappropriate storage of samples and reagents at the laboratory level is mostly due to the discontinuity of electricity supply. Altogether, these factors lead to the limited submission of samples for testing, which in turn reduces the surveillance capacity in the poorest countries (41). In such contexts, the availability of diagnostic solutions that require only basic storage conditions would be highly beneficial. Great efforts have been made to develop and commercialize this kind of product for AIV and RABV detection, which are however mostly based on point-of-care (POC) antigenic tests whose sensitivity is lower than that of PCR-based methods (44–50). An attractive alternative is represented by freeze-dried reagents for PCR-based applications, which promise to offer the sensitivity of molecular methods with less strict requirements for reagent preservation (51). While the application of these reagents is still unexplored for RABV detection, a number of independent studies have preliminarily demonstrated the suitability of lyophilized formulations for the molecular diagnosis of avian influenza (44, 52–56). However, these studies neither assessed the performance of the lyophilized reagents in the actual conditions faced by low-income countries nor were they restricted to the evaluation of screening methods only, neglecting downstream subtyping assays. To explore more in depth the potentiality of freeze-dried amplification kits with basic storage requirements, we compared a commercial lyophilized reagent to liquid master mixes employing real-time RT-PCR (qRT-PCR) and end-point RT-PCR methods for the detection and typing of avian influenza and rabies viruses. The suitability of the lyophilized reagent in replacing current liquid master mixes was further assessed by determining its actual thermostability under stressful environmental conditions, as well as through an inter-laboratory reproducibility study that involved four Central Veterinary Laboratories (CVLs) located in Sub-Saharan Africa.

## MATERIALS AND METHODS

### Selection of freeze-dried kits and molecular protocols

We performed an inventory analysis of commercial freeze-dried master mixes meeting the following criteria: i) one-step RT-PCR/qRT-PCR for the detection of RNA targets; ii) suitability for TaqMan probe-based qRT-PCR assays; iii) adaptability to custom oligonucleotides. The performance of the selected product was extensively compared to that of standard liquid reagents employed in reference qRT-PCR and end-point RT-PCR methods for AIV and RABV detection and typing (Table 1) (30, 31, 36, 57–65), the majority of which have been widely adopted also in the CVLs taking part in this study. For analytical sensitivity, repeatability, and thermostability tests, viral RNA was purified from viral isolates with the QIAamp Viral RNA kit (Qiagen). For diagnostic sensitivity and inter-laboratory reproducibility, the NucleoSpin RNA kit (Macherey-Nagel) was used. When performing the protocols described in (59, 63), six microliters of the exogenous control intype IC-RNA (Indical) was added to the lysate during RNA isolation.

**TABLE 1:** AIV and RABV molecular protocols employed to compare lyophilized and liquid master mix reagents^*a*^

Type of assay	Denomination	Target	Reference	Liquid reagent	Assessed parameters
qRT-PCR	AIV#1	AIV M gene	(59, 63)	AgPath-ID One-Step RT-PCR Reagents (Applied Biosystems)	ASe, DSe, and S
	AIV#2	AIV M gene	(60)	QuantiTect Multiplex RT-PCR kit (Qiagen)	ILR
	H5#1	H5 HA2	(61)	One-Step RT-PCR kit (Qiagen)	ASe, DSe, ILR, and S
	H7	H7 HA2	(62, 64)	QuantiTect Probe RT-PCR kit (Qiagen)	ASe
	H9	H9 HA2	(65)	AgPath-ID One-Step RT-PCR Reagents (Applied Biosystems)	ASe and R
	RABV#1	3’UTR/N gene	(58)	AgPath-ID One-Step RT-PCR Reagents (Applied Biosystems)	ASe, DSe, R, ILR, and TOC
	β-actin^*b*^	β-actin mRNA	(58)	AgPath-ID One-Step RT-PCR Reagents (Applied Biosystems)	R and ILR
	RABV#3	3’UTR/N gene	(66)	AgPath-ID One-Step RT-PCR Reagents (Applied Biosystems)	TOC
End-point RT-PCR	H5#2	H5 HA1/HA2	(67)	One-Step RT-PCR kit (Qiagen)	ASe and DSe
	RABV#2	N gene	(57)	One-Step RT-PCR kit (Qiagen)	ASe, DSe, R, and ILR

^*a*^ ASe: analytical sensitivity; DSe: diagnostic sensitivity; R: repeatability; ILR: inter-laboratory reproducibility; S: stability; TOC: template/oligo complementarity assessment.

^*b*^ Endogenous internal control assay coupled with the RABV#1 assay and performed on a separate reaction to assess the integrity of the extracted RNA.

Reaction mix compositions and optimized amplification protocols are described in detail in the S1 file. All qRT-PCR assays were performed on a CFX96 Touch Deep Well Real-Time PCR Detection System (Biorad) and analyzed with the CFX Maestro Software. End-point RT-PCR protocols were carried out on the S1000 Thermal Cycler (Biorad), and the amplification products were visualized with the QIAxcel Advanced System (Qiagen). Sanger sequencing downstream RT-PCR was performed using the Genetic Analyzer 3500xL Dx (Applied Biosystems).

### Analytical sensitivity (ASe)

Analytical sensitivity was assessed by testing ten-fold dilution series of synthetic RNA and viral isolates prepared in molecular-grade water and PBS with antibiotics/antimycotics (PBSa), respectively (Table 2). Synthetic RNA was produced from plasmid templates as described elsewhere (66). Each dilution was tested in three replicas employing the selected lyophilized reagent, as well as the liquid master mixes for comparison. The limit of detection (LoD) was determined as the highest dilution at which all tested replicates yielded positive results. For end-point RT-PCR, positive results were confirmed by Sanger sequencing.

**TABLE 2:** Samples used for analytical sensitivity

Virus	Strain description	Accession number^*a*^	Matrix
AIV	A/chicken/Togo/17RS1021-1/2017 /H5N1 HPAI 2.3.2.1 c	EPI_ISL_273845	Allantoic fluid
	A/chicken/Italy/1279/99 /H7N1 LPAI	DQ090029.1	
	A/chicken/Nigeria/RV-C11-15OR_19VIR8424-15/2019 /H9N2 G1	EPI_ISL_14768087	
RABV	RABV lineage Africa 2 (Phylogroup I)	MK471246	Synthetic RNA
Non-RABV lyssaviruses	Duvenhage virus, DUVV (Phylogroup I)	EU293120	Synthetic RNA
	Mokola virus, MOKV (Phylogroup II)	EU293118	
	Lagos bat virus/lineage c, LBV (Phylogroup II)	EU293108	
	Ikoma lyssavirus, IKOV (Phylogroup III)	NC_018629	

^*a*^ Global Initiative on Sharing All Influenza Data (GISAID) EpiFlu database or NCBI database.

### Repeatability within run and between days

Repeatability of Cq values obtained with the lyophilized reagent was assessed at two different concentrations of avian influenza A/chicken/Nigeria/RV-C11-15OR_19VIR8424-15/2019 (dilutions tested: 10^3.5^ and 10^1.5^ EID_50_/100 μL) and MOKV synthetic RNA (dilutions tested: 10^4^ and 10^2^ RNA copies/μL) employing the H9 and RABV#1 assays, respectively. Each dilution was tested in triplicate on 2 different days. The coefficient of variation (CV) of Cq values within run and between days was calculated to estimate the repeatability.

### Diagnostic sensitivity (DSe)

Diagnostic sensitivity was assessed by testing avian influenza and rabies-positive specimens retrieved from the IZSVe repository employing the freeze-dried reagent. They consisted of 24 bird samples (cloacal and oropharyngeal swabs and pool of organs) representing H5 HPAI viruses of clades 2.3.4.4b and 2.3.2.1 c and of 28 RABV infected brains representing the Cosmopolitan (Eastern Europe, Western European, and Africa 1 clades), Africa 2, Africa 3, Arctic-like, and American indigenous lineages (Tables S1 and S2).

Swab heads were cut, placed in 750–1,000 µL PBSa, and vortexed. Bird organs were diluted 1:4 wt/vol with PBSa and homogenized with the TissueLyser II (Qiagen) at 30 Hz for 3 min, while brain samples were homogenized in PBS (1:10 wt/vol) with a glass rod. Homogenized tissues were subsequently clarified to obtain the supernatant. RNA was extracted from 100 µL of the swab suspension or tissue supernatant. For avian influenza testing, the intype IC-RNA (Indical) was co-amplified with the viral genome (assay AIV#1) to ensure correct RNA extraction and verify the absence of PCR inhibition. For RABV diagnostics, specimen conformity was verified by qRT-PCR targeting endogenous ß-actin mRNA (58). For comparison, all samples were also processed with standard liquid reagents. Data were plotted using Prism 9.3.1 (GraphPad, San Diego, CA, USA), and the Spearman’s rank correlation coefficient (*r*) of the Cq obtained with the lyophilized and liquid reagents was calculated. DSe and its exact binomial confidence interval (95% level) were assessed for each protocol using both reagents, and the comparability of sensitivity was assessed by the two-sample test of proportions. A *P*-value < 0.05 was considered significant.

### Stability verification of the lyophilized reagent

In order to verify the actual stability of the lyophilized reagent, we tested an H5N1 HPAI 2.3.2.1 c isolate (A/chicken/Togo/17RS1021-1/2017) at high, medium, and low concentrations (10^4.5^, 10,^1.5^ and 10^0.5^ EID_50_/100 μL, respectively) with the AIV#1 and H5#1 assays after storing the freeze-dried kit under the following conditions: +4°C for 2 months, room temperature for 9 months, and 30°C for 10 days. Storage at +4°C and room temperature not exceeding 9 months is guaranteed by the manufacturer, while reagent incubation at 30°C prior to use was evaluated to mimic lapses that might occur during the reagents’ transportation in the absence of controlled temperature. Samples were tested also with liquid reagents as a gold-standard reference following the manufacturer’s instruction for storage. The Mann-Whitney test was applied to verify any significant difference in terms of Cq values among the different storage conditions using Prism 9.3.1 (GraphPad, San Diego, CA, USA). A *P*-value < 0.05 was considered significant.

### Deployment of the lyophilized reagent through an inter-laboratory reproducibility study

An inter-laboratory reproducibility study was organized to deploy the lyophilized reagent for the molecular diagnosis of AI and rabies to CVLs of Sub-Saharan Africa. Four laboratories were invited to take part in the study, namely, Laboratoire Central Vétérinaire (Mali), Laboratoire National d’Elevage et de Recherches Vétérinaires (Senegal), Laboratoire National Vétérinaire Yaoundé (Cameroon), and Laboratoire Central de Diagnostic Vétérinaire de Conakry (Guinea). At the time of the exercise, participating laboratories were routinely diagnosing AI and rabies through qRT-PCR and end-point RT-PCR, respectively. They all declared to have never applied qRT-PCR in the diagnosis of rabies.

Participants were provided with (i) Standard Operating Procedures (SOPs) for AIV#2, H5#1, H5#2, RABV#1, β-actin, and RABV#2 assays, to be performed with both lyophilized and liquid reagents; (ii) nucleic acid extraction kit, amplification kits, and oligonucleotides necessary to perform the exercise; and (iii) panels of blind samples for avian influenza and rabies testing. Concomitantly to the shipment, an online meeting was arranged to provide appropriate instructions. At the end of the exercise, the laboratories were invited to submit results using a standardized data reporting form.

Test samples (RABV panel: *n* = 13; AIV panel: *n* = 12) comprising negative and positive specimens for the assessed targets were prepared using PrimeStore Molecular Transport Medium (MTM) (Longhorn Vaccines and Diagnostics, MD, USA) and appropriately blind-coded. Quality control tests (i.e., homogeneity and stability) were performed according to the ISO/IEC 17043:2010 and ISO 13528:2022 and using both liquid and freeze-dried reagents. Full panel compositions and expected results are detailed in Tables S3 and S4.

### Data analyses of the inter-laboratory reproducibility study

For data analysis, laboratories were anonymized with unique letters (A–E).

First, we assessed whether the lyophilized reagent was able to correctly identify positive and negative samples for all the targets considered. In detail, we compared the results obtained by all five laboratories (four CVLs and the Reference Laboratory—RL, hosted by the IZSVe) with the freeze-dried reagent to the results obtained using the reference liquid master mixes by the RL. For each participant, we also evaluated the impact the lyophilized reagent could have on the target identification by comparing the results obtained at a laboratory level and between participants (freeze-dried vs the liquid reagents). In both cases, the McNemar test was applied with matched pairs of dichotomous results (positive and negative) for all tested protocols but the β-actin assay, for which the Stuart Maxwell test was used (k categories: positive, negative, and weakly positive). Cohen’s Kappa coefficient was also calculated and interpreted according to the Landis and Koch (1977) scale (< 0: no agreement; 0–0.20: slight agreement; 0.21–0.40: fair agreement; 0.41–0.60: moderate agreement; 0.61–0.80: substantial agreement; 0.81–0.99 almost perfect agreement; 1 perfect agreement).

Second, we assessed whether the differences between Cq values obtained with the lyophilized and liquid reagents were statistically significant through Bland-Altman plots. The normal distribution of Cq values was verified with the Shapiro-Wilk test. In case of the normal distribution, the one-sample *t*-test was performed to assess whether the mean bias was significantly different from 0, and the limits of agreement were calculated as mean bias ±2 standard deviation. In case of non-normal Cq distribution, the limits of agreement were calculated as 2.5% and 97.5% percentiles of the difference distribution, and the significance of the median bias was tested using the Wilcoxon test. The mean or median bias, limits of agreement, and the line of equality between liquid and lyophilized reagents were plotted into graphs.

Ultimately, we assessed the correct implementation of the qRT-PCR assay for rabies diagnosis at the four enrolled CVLs, employing RABV#1 and β-actin qRT-PCRs. In this context, statistical analysis was performed according to the number of “correct” results, i.e., answers in agreement with the reference values assigned by the RL. All results were statistically analyzed using either the McNemar or Stuart Maxwell tests and the Cohen’s Kappa coefficient determination, as described above.

*P*-value < 0.05 was considered statistically significant for all data analysis. Statistical software R version 4.2.1 was used to perform the statistical analyses.

## RESULTS

### The Qscript lyo 1-step (Quantabio) was selected for analytical performance evaluation compared to standard liquid reagents

At the time of the study, we identified six different freeze-dried one-step qRT-PCR/end-point RT-PCR commercial products: the Qscript lyo 1-step (Quantabio, MA, USA), the Takyon Dry One-Step RT Probe MasterMix No Rox (Eurogentec, Belgium), the 5X CAPITAL qRT PCR Probe Master Mix lyophilized (Biotech rabbit, Germany), the SCRIPT RT-qPCR ProbesMaster Lyophilisate (Jena Science, Germany), the Lyophilized One Step qRT PCR (Biotechne SRL, Italy), and the ViPrimePLUS Lyophilized One Step qRT-PCR Master Mix (Vivanti, Malaysia). The main features of the above kits are detailed in Table S5.

All multi-reaction kits requiring reconstitution of the entire lyophilized product prior to the reaction assembly were excluded due to the need to freeze the unused reagent after rehydration. In contrast, reagents pre-aliquoted as lyospheres in 200 μL tubes for single-reaction assembly were considered for further evaluation as they i) do not require storage at −20°C once reconstituted, ii) reduce the risk of cross-contamination, iii) allow higher repeatability by minimizing the effect of pipetting errors of the master mix, and iv) permit using the tubes provided with no need to transfer the reaction mix, thus simplifying the reaction assembly. The Qscript lyo 1-step (Quantabio) and SCRIPT RT-qPCR ProbesMaster Lyophilisate (Jena) met such requirements, and their ASe was preliminarily assessed using the RABV#1 assay (58). The Qscript lyo 1-step (Quantabio) proved to be more sensitive (data not shown) and was selected for subsequent evaluation.

For all qRT-PCR and end-point RT-PCR assays assessed and applied to AIV and RABV samples, the Qscript lyo 1-step kit showed the same LoD as the liquid master mixes. The internal control implemented for the AIV#1 assay provided conforming results in all cases. In contrast, lower analytical sensitivity was obtained for the H5#2 assay, which is based on an end-point RT-PCR and yields amplification products of 308 bp (LoD increased by 1 log) as well as for the RABV#1 and RABV#2 assays applied to non-RABV lyssaviruses (LoD increased up to 2 logs depending on the species) (Table 3).

**TABLE 3:** Analytical sensitivity (ASe) of the molecular protocols performed with the Qscript lyo 1-step kit and the liquid reagents^*a*^

Assay	Sample	LoD (mean Cq ±SD)	
		Liquid reagents	Lyophilized reagent
AIV#1	H5N1	10^0.5^ (35.8 ± 0.4)	10^0.5^ (35.0 ± 0.7)
H5#1	H5N1	10^0.5^ (36.0 ± 0.7)	10^0.5^ (35.6 ± 0.3)
H7	H7N1	10^0.5^ (35.2 ± 0.4)	10^0.5^ (37.8 ± 1.4)
H9	H9N2	10^0.5^ (31.7 ± 1.3)	10^0.5^ (34.1 ± 1.2)
H5#2	H5N1	**10^-0.5^**	**10^0.5^**
RABV#1	RABV	10 (33.8 ± 0.5)	10 (33.4 ± 0.1)
	DUVV	**10^2^** (35.0 ± 0.5)	**10^3^** (30.9 ± 0.1)
	MOKV	10^2^ (33.8 ± 0.3)	10^2^ (34.2 ± 0.3)
	LBV	**10^3^** (33.4 ± 0.1)	**10^5^** (33.2 ± 0.3)
	IKOV	10^5^ (34.0 ± 0.1)	10^5^ (35.2 ± 0.2)
RABV#2	RABV	10^2^	10^2^
	DUVV	**10^3^**	**10^4^**
	MOKV	**10^3^**	**10^5^**
	LBV	10^7^	10^7^
	IKOV	10^3^	10^3^

^*a*^ LoD values are expressed as EID_50_/100 μL or RNA copies/μL, according to the matrix employed. LoD values qualitatively different between the liquid and lyophilized reagents are highlighted in bold. For qRT-PCR assays, mean Cq and standard deviation values are reported in brackets.

The Qscript lyo 1-step (Quantabio) yielded repeatable results over the assays and virus concentration tested, with a CV of Cq values ≤ 0.04.

### The lyophilized reagent is suitable for clinical sample testing

**Fig 1:**
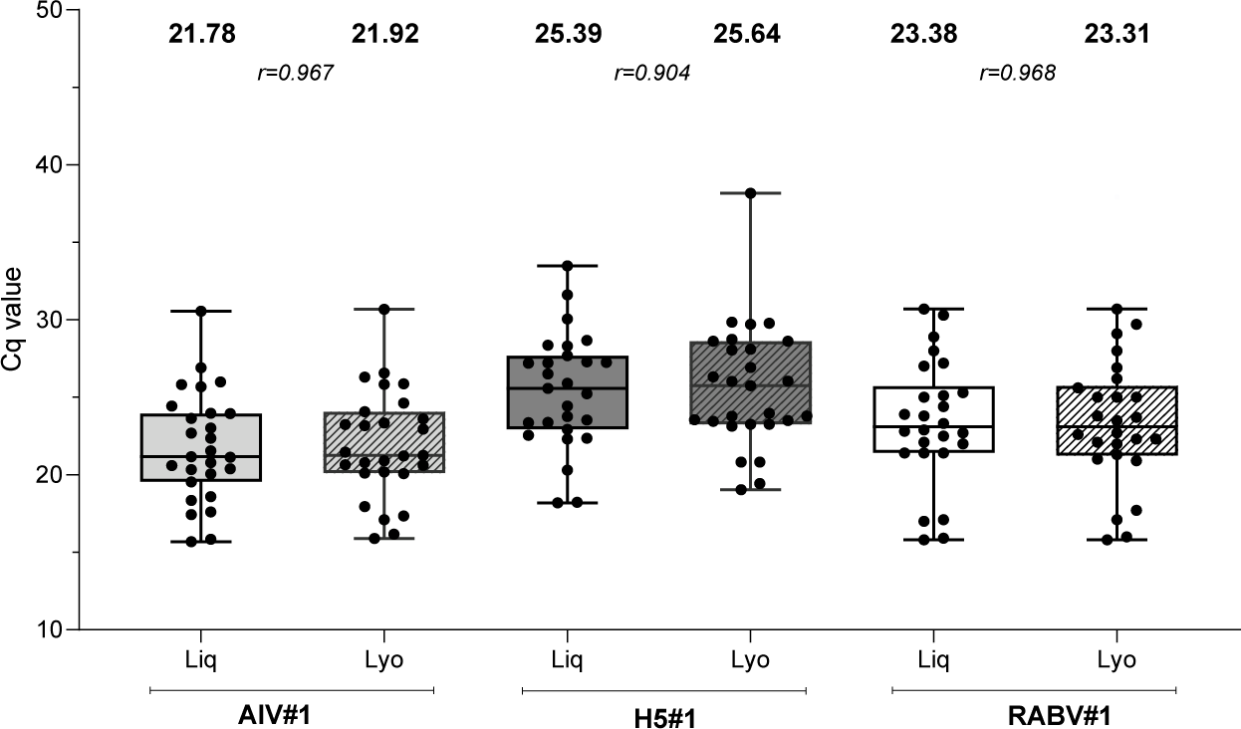
Diagnostic sensitivity of AIV#1, H5#1, and RABV#1 assays performed either with liquid and lyophilized reagents. Dots represent individual Cq values. Mean of Cq values for all samples at the different tested conditions (in bold) and Spearman's rank correlation coefficient (R) between the liquid and the lyophilized reagents are reported. Liq: liquid reagent; Lyo: lyophilized reagent.

All but one AIV-H5 positive sample representing different bird species, geographic origins, and sample matrices were correctly identified by the AIV#1 and H5#1 assays employing the Qscript lyo 1-step (Quantabio), resulting in 96.55% DSe (Table 4). Most likely, sample 17RS115-4 was missed because of low viral load (Table S1). The RABV#1 and RABV#2 assays employing the Qscript lyo 1-step (Table 4) correctly identified (100% DSe) all the tested RABV positive field samples. Overall, no significant differences were observed between lyophilized and liquid reagents, neither for avian influenza nor for rabies, with a high correlation of Cq values (*r* ≥ 0.904) (Fig. 1).

**TABLE 4:** Diagnostic sensitivity (DSe) of molecular protocols performed with the Qscript lyo 1-step kit compared to the liquid reagents^*a*^

Assay	No. of samples tested	DSe (95% CI)	
		Liquid reagents	Lyophilized reagent
AIV#1	24	100 (88.05–100)^*b*^	96.55 (82.23–99.91)
H5#1		96.55 (82.23–99.91)	96.55 (82.23–99.91)
RABV#1	28	100 (87.23–100)^*b*^	100 (87.23–100)^*b*^
RABV#2		100 (87.23–100)^*b*^	100 (87.23–100)^*b*^

^*a*^ DSe is expressed as percentage with 95% confidence interval.

^*b*^ one-side – 97.5% CI.

### The performance of the lyophilized reagent might be affected by primer-template complementarity

**Fig 2:**
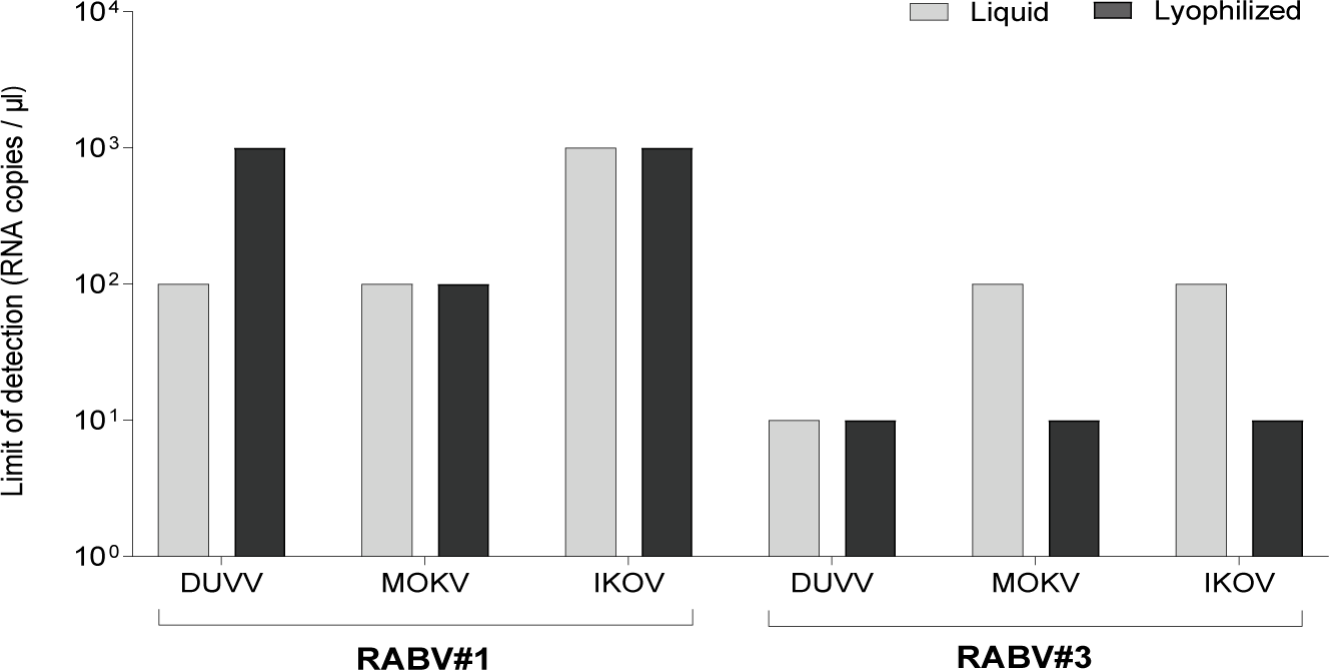
Limit of detection of the RABV#1 and the RABV#3 assays employing the lyophilized reagent Qscript lyo 1-step (Quantabio) on serial dilutions of synthetic RNA from DUVV (phylogroup I), MOKV (phylogroup II), and IKOV (phylogroup III).

After observing a decreased ASe of the Qscript lyo-1 step (Quantabio) compared to the liquid reagents when testing non-RABV lyssaviruses, we investigated more in depth the effect of oligonucleotide-template complementarity on the performance of the lyophilized reagent. To this aim, we applied the Qscript lyo-1 step (Quantabio) to the primers and probe from the (n) LN34 assay (RABV#3), which has been recently developed to improve the coverage against divergent lyssaviruses (66). LoD was re-assessed on serial dilutions of synthetic RNA from DUVV (phylogroup I), MOKV (phylogroup II), and IKOV (phylogroup III). Contrary to what was observed for RABV#1, the ASe of RABV#3 (66) combined with the Qscript lyo-1 step (Quantabio) showed the same (DUVV) or improved (MOKV and IKOV) sensitivity compared to the liquid reagent (Fig. 2; Table S6). These results suggest that the Qscript lyo 1-step (Quantabio) is more affected by suboptimal primer-template complementarity, compared to liquid mastermixes.

### The lyophilized reagent shows optimal stability even when stored at 30°C for short periods

**Fig 3:**
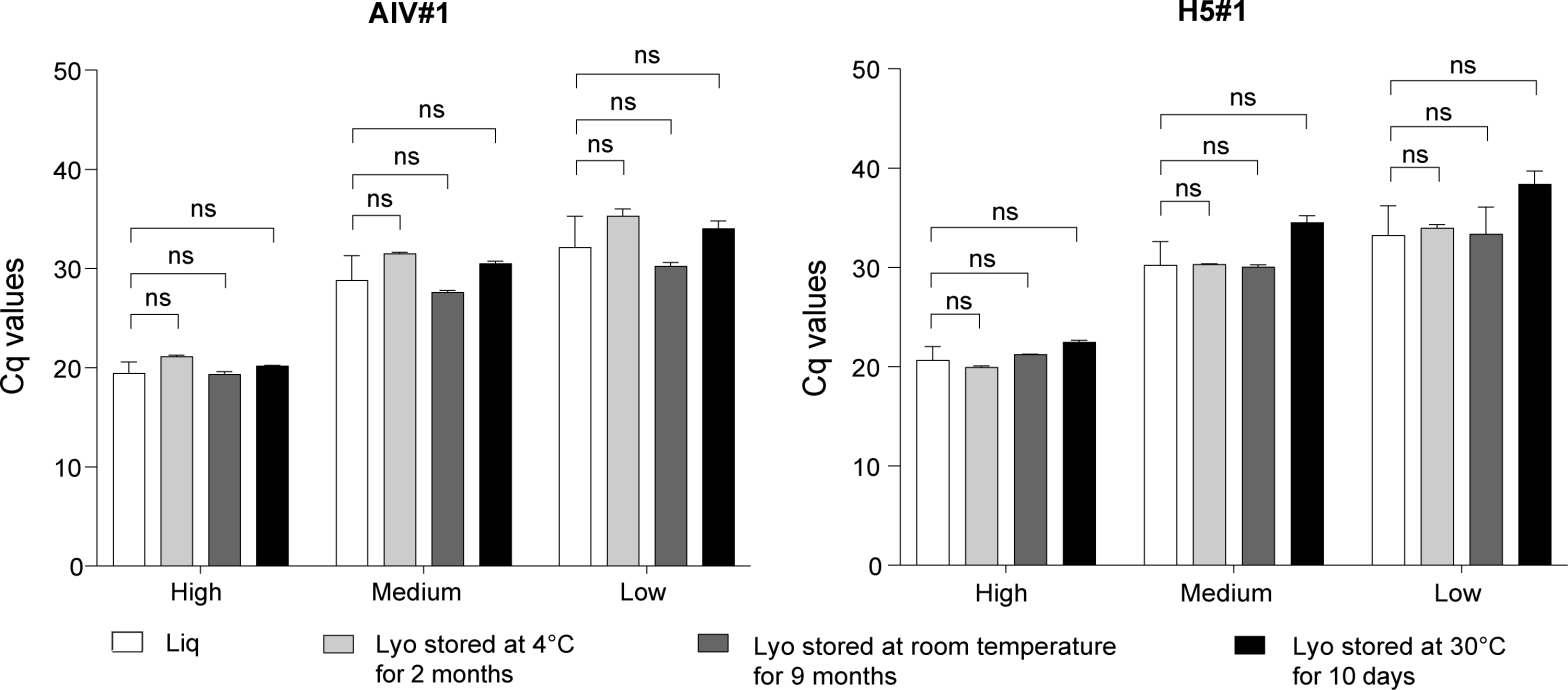
Stability of the lyophilized kit under different storage conditions. The Mann-Whitney test was applied to assess differences between mean Cq values of the reference (liquid reagents; Liq) and lyophilized reagent (Lyo) Qscript lyo 1-step. High, medium, and low concentrations of H5N1 (A/chicken/Togo/17RS1021-1/2017) were employed. ns: not significant.

To evaluate the effect of environmental stress conditions that might occur during reagent shipment or in the laboratory, the lyophilized kit was stored at different temperatures before use. Data obtained (Fig. 3; Table S7) indicate that the performance of the Qscript lyo 1-step (Quantabio) is comparable to that of the liquid reagents over the tested range of temperatures and incubation times, without any loss of sensitivity, as assessed by the LoD. These comprise incubation for 10 days at 30°C, a scenario that might occur upon delayed reagent delivery or inadequate storage due to electricity blackouts at the laboratory level. Notably, the use of the Qscript lyo 1-step (Quantabio) stored for 10 days at 30°C and for 9 months at room temperature showed slightly higher Cq values with respect to the lyophilized reagent stored under optimal conditions and to liquid reagents, although differences were not statistically significant (*P* > 0.05).

### The lyophilized reagent can be deployed in Sub-Saharan diagnostic laboratories for AIV and RABV molecular testing

The results obtained by all the laboratories taking part in the exercise and using the Qscript lyo 1-step kit (Quantabio) demonstrated perfect or almost perfect agreement with the reference results obtained by the RL (Cohen’s-Kappa 0.921–1) using liquid reagents, with similar Cq values (McNemar/Stuart Maxwell test *P*-value = 0.157–1) (Table 5).

**TABLE 5:** Agreement between the qualitative results of the Qscript Lyo-1 step kit (Quantabio) and the reference results^*a*^

Assay	Cohen’s Kappa^*b*^	McNemar/Stuart Maxwell test *P*-value
AIV#2	0.963	0.317
H5#1	0.931	0.157
RABV#1	0.937	1
β-actin	0.921	0.223
RABV#2	1	n.a.^*c*^

^*a*^ Data from all participants obtained with the Qscript lyo 1-step kit (Quantabio) were compared to the reference results obtained by the RL that employed standard liquid master mixes.

^*b*^ *P*-value < 0.001.

^*c*^ n.a.: not applicable.

When assessing the results obtained by each laboratory, statistical analyses revealed a perfect or almost perfect agreement between the Qscript lyo 1-step (Quantabio) and the liquid reagents for all the assays, except for β-actin in laboratory B, and RABV#1 and AIV#2 in laboratory D, which showed a substantial agreement (Table 6). The McNemar or Stuart Maxwell test demonstrated that there was no significant difference in the number of “correct” results (Table 6).

**TABLE 6:** Agreement between Qscript Lyo-1 Step Kit (Quantabio) and liquid reagent qualitative results for each laboratory and overall^*a*^

Assay	Test	Value	Laboratory					
			A	B	C	D	E	All labs
AIV#2	McNemar	*P-*value	n.a.	n.a.	n.a.	0.317	n.a.	1
	Cohen’s kappa	*Kappa*	1	1	1	0.800	1	0.925
		*P-*value	< 0.001	< 0.001	< 0.001	0.002	< 0.001	< 0.001
H5#1	McNemar	*P-*value	0.317	0.317	n.a.	n.a.	n.a.	0.157
	Cohen’s kappa	*Kappa*	0.824	0.824	1	1	1	0.931
		*P-*value	0.002	0.002	< 0.001	< 0.001	< 0.001	< 0.001
RABV#1	McNemar	*P-*value	n.a.	n.a.	n.a.	1	n.a.	0.317
	Cohen’s kappa	*Kappa*	1	1	1	**0.675**	1	0.872
		*P-*value	< 0.001	< 0.001	< 0.001	0.007	< 0.001	< 0.001
β-actin	Stuart Maxwell	*P-*value	n.a.	0.367	0.606	n.a.	n.a.	0.007
	Cohen’s Kappa	*Kappa*	1	**0.745**	0.851	1	1	**0.723**
		*P-*value	< 0.001	< 0.001	< 0.001	< 0.001	< 0.001	< 0.001
RABV#2	McNemar	*P-*value	n.a.	n.a.	n.a.	n.p.	n.a.	n.a.
	Cohen’s kappa	*Kappa*	1	1	1	n.p.	1	1
		*P-*value	< 0.001	< 0.001	< 0.001	n.p.	< 0.001	< 0.001

^*a*^ n.a: not applicable; n.p.: analysis not performed. Cohen's kappa < 0.750 is highlighted in bold.

**Fig 4:**
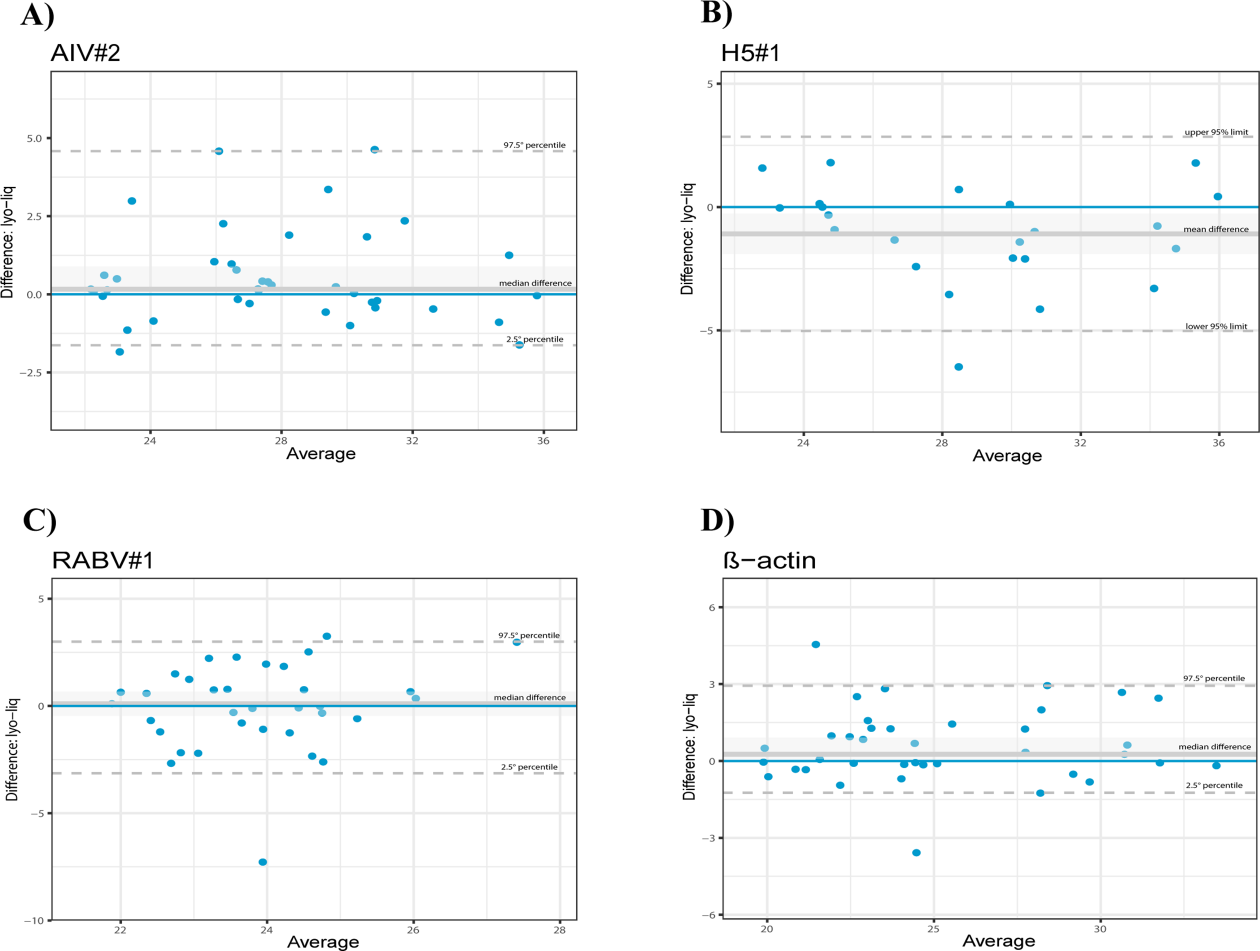
The Bland-Altman plots show differences between Ct values of liquid reagents vs the AIV#2, H5#1, RABV#1, and ẞ-actin rRT-PCR protocols run with the Qscript lyo 1-step. The blue lines represent perfect agreement. The gray lines represent the mean or median bias (according to the satisfaction or not of the normality requirement) between the lyophilized ("lyo") and liquid ("liq") reagents. The gray dotted lines represent the upper and lower limits of agreement. (**A**) AIV#2: The differences between the two methods are within the −1.630 and 4.582 range, and the two methods may be used interchangeably. The median bias of 0.165 was not significantly different from zero (Wilcoxon test *P*-value = 0.082, the line of equality is within the confidence interval of the median difference); (**B**) H5#1: The differences between the two methods are within the −5.024 and 2.850 range. The mean bias −1.087 was significantly different from 0 (*t*-test test *P*-value = 0.0165, the line of equality is not within the confidence interval of the mean difference). Bland-Altman diagram shows a substantial systematic difference between the two methods, resulting in better performances of the lyophilized reagent; (**C**) RABV#1: The differences between the two methods are within the −3.130 and 2.998 range; the two methods can be used interchangeably. The median bias of 0.103 was not significantly different from zero (Wilcoxon test *P*-value = 0.774, the line of equality is within the confidence interval of the median difference); (**D**) ẞ-actin: The differences between the two methods are within the −1.240 and 2.934 range. The mean bias −0.260 was significantly different from 0 (Wilcoxon test *P*-value = 0.027, the line of equality is not within the confidence interval of the median difference). The Bland-Altman diagram shows a substantial systematic difference between the two methods leading to worse performances with lyophilized reagents.

Perfect or almost perfect agreement between aggregated data obtained from all laboratories with the lyophilized reagent compared to liquid master mixes was calculated for all assays, but β-actin, which showed a substantial agreement (Cohen’s kappa 0.723; Stuart Maxwell test *P*-value = 0.007). The Bland-Altman plots revealed no significant differences in terms of Cq values between the Qscript lyo 1-step kit (Quantabio) and the liquid reagents for AIV#1 and RABV#1 assays, while Cq values decreased for the lyophilized reagent when applying the H5#1 assays, meaning that freeze-dried and liquid master mixes are interchangeable. In contrast, an increase in Cq values was observed for the β-actin assay (Fig. 4).

### Rabies qRT-PCR was successfully implemented in Sub-Saharan CVLs

As the CVLs had declared no prior experience in implementing RABV#1, and more in general qRT-PCR assays to diagnose rabies, we compared the results obtained using the reference liquid master mix at their laboratories with those from the RL. Perfect or almost perfect agreement was observed between the RL and the enrolled CVLs, with only one exception, for which substantial/fair agreement was achieved for the RABV#1/β-actin assays, respectively (Table 7).

**TABLE 7:** Implementation of the qRT-PCR for rabies diagnosis in Sub-Saharan laboratories^*a*^

Assay	Test	Value	Laboratory				
			A	B	C	D	All labs
RABV#1	McNemar	*P-value*	0.157	n.a.	n.a.	n.a.	0.157
	Cohen’s kappa	*Kappa*	**0.698**	1	1	1	0.920
		*P-value*	0.004	< 0.001	< 0.001	< 0.001	< 0.001
β-actin	Stuart Maxwell	*P-value*	n.a.	n.a.	n.a.	n.a.	0.030
	Cohen’s kappa	*Kappa*	**0.435**	0.851	1	0.851	0.753
		*P-value*	0.002	< 0.001	< 0.001	< 0.001	< 0.001

^*a*^ n.a.: not applicable. Cohen's kappa < 0.750 is highlighted in bold.

## DISCUSSION

Unreliable electricity provision as well as supply chain issues under refrigeration or frosting are recognized among the most significant factors that impact the ability to perform accurate lab work in Sub-Saharan Africa. In these scenarios, lyophilized reagents for molecular testing appear as effective solutions to overcome such constraints as they are developed to combine higher stability and temperature resilience, simpler transportation and storage, space efficiency, ease of use, higher repeatability and reproducibility, and, ultimately, cost-effectiveness.

Among the different freeze-dried formulations commercially available, we selected the Qscript lyo 1-step (Quantabio) for its single-reaction format that offers numerous advantages in terms of conservation, reaction assembly, reliability, and performance. A previous work has preliminarily demonstrated the utility of this product for AIV screening by qRT-PCR (56). However, the current study provides the first comprehensive evaluation of its functionality throughout the entire diagnostic workflow, starting from the nucleic acid extraction to AIV subtyping by both qRT-PCR/RT-PCR and Sanger sequencing, and has further extended its validation to rabies diagnosis. Importantly, our data indicate that the replacement of liquid reagents with the Qscript lyo 1-step (Quantabio) in a wide range of qRT-PCR and end-point protocols produces repeatable results without significantly affecting the sensitivity for the majority of the targets evaluated, as assessed by LoD determination and diagnostic performance on positive clinical samples. However, it is worth noting that the end-point RT-PCR H5#2 yielded an LoD improved by 1 log when using the liquid reagents. It can be assumed that the Qscript lyo 1-step (Quantabio) sensitivity slightly decreases when longer targets are amplified, as a result of suboptimal reaction kinetics that are specific to each protocol and warrant evaluation on a case-by-case basis. Another condition that most likely affected the functionality of the lyophilized reagent is the suboptimal oligonucleotide complementarity (56, 68). Indeed, we observed a decrease in sensitivity when we applied the Qscript lyo 1-step kit (Quantabio) to DUVV and LBV with the RABV#1 assay. Notably, the detection of divergent lyssaviruses was restored with the RABV#3 assay, a method specifically designed to widen assay inclusivity and sensitivity by enhancement of the primer-template complementarity (66). Although the authors acknowledge that further evaluation is necessary to recommend the RABV#3 in place of the RABV#1 assay using the lyophilized reagent, our results support the hypothesis that the Qscript lyo 1-step kit (Quantabio) performance depends, at least in part, on oligonucleotide complementarity toward the target region and underlines the need for a careful evaluation of the diagnostic method well before implementing the use of lyophilized reagents.

Importantly, to accomplish the deployment of AI and RABV diagnostic assays based on the use of the lyophilized reagent, our validation scheme included an inter-laboratory reproducibility exercise that engaged four CVLs located in Sub-Saharan Africa. Data submitted by the participants confirmed a perfect or almost perfect agreement with the expected results and demonstrated that the implementation of the lyophilized reagent in place of liquid formulations allowed the correct identification of AIV and RABV in most cases, resulting in highly reproducible results among different laboratories. Considering rabies diagnosis, it is worth noting that the four CVLs had no prior experience with the RABV#1 assay or with any other qRT-PCR protocol for the diagnosis of rabies, but were anyway able to successfully implement such techniques at their facilities. This lack of experience may partly explain the suboptimal results observed for the β-actin assay using both the liquid and the lyophilized master mixes. Although improvable, the successful acquisition of the qRT-PCR protocol by the four participating CVLs represents a significant achievement for surveillance and confirms that molecular assays can be easily implemented in tropical regions (37), specifically in veterinary laboratories that have been trained and supported to enable early diagnosis of other priority animal diseases (69–72). Notably, the participating laboratories reported no false positives during the inter-laboratory exercise, further confirming the ease of use and reliability of the lyophilized reagent format. Furthermore, the simultaneous application of liquid reagents throughout the trial allowed a quantitative evaluation of the Qscript lyo 1-step kit (Quantabio) performance applied to qRT-PCR assays, which revealed an irrelevant shift of Cq values (as assessed by intra- and inter-laboratory comparisons) and demonstrated that the freeze-dried and liquid master mixes can be used interchangeably.

Another key feature that further confirms the feasibility of using the lyophilized reagent in Sub-Saharan regions is its thermal stability. According to the manufacturer’s recommendations, the Qscript lyo 1-step kit (Quantabio) can be stored at room temperature (21°C–23°C) for up to 9 months. However, temperature can vary during transportation, and storage is barely controlled in tropical and subtropical areas. We therefore challenged the lyophilized kit by incubation at 30°C for 10 days to mimic the absence of cold-chain maintenance (37). The Qscript lyo 1-step kit (Quantabio) proved to be stable under such conditions and at the same time resilient in case of electricity discontinuation at laboratory facilities or delayed intercontinental parcel delivery at uncontrolled temperatures.

In conclusion, our study demonstrates that lyophilized reagents can replace liquid formulations for AI and RABV molecular diagnosis and provide reliable results under real Sub-Saharan laboratory settings. The successful application of the Qscript lyo 1-step kit (Quantabio) on a variety of assays and targets suggests that its use has the potential to be extended to the molecular diagnosis of other relevant veterinary and zoonotic diseases in remote areas characterized by poor transportation and storage infrastructures. However, on a larger scale, the implementation of lyophilized reagents requires accurate optimization of the amplification conditions and a thorough performance evaluation of the selected protocols.

## SUPPLEMENTAL MATERIAL

Supplemental tables jcm.00080-25-s0001.docx Tables S1 to S7.

ASM does not own the copyrights to Supplemental Material that may be linked to, or accessed through, an article. The authors have granted ASM a non-exclusive, world-wide license to publish the Supplemental Material files. Please contact the corresponding author directly for reuse.
