# Supplementary material for: Validation and multi-site deployment of a lyophilized qRT-PCR reagent for the molecular diagnosis of avian influenza and rabies in Sub-Saharan African regions
Source: J Clin Microbiol. 2025 Jul 1;63(8):e00080-25. doi: 10.1128/jcm.00080-25 (PMC12345159; doi:10.1128/jcm.00080-25)
Supplement: Supplemental tables — Tables S1 to S7. [file jcm.00080-25-s0001.docx]

**Supplementary tables to: Validation and multi-site deployment of a lyophilized qRT-PCR reagent for the molecular diagnosis of avian influenza and rabies in Sub-Saharan African regions**

**Petra Drzewnioková^1,2^, Irene Brian^1^, Marzia Mancin^1^, Andrea Fortin^1^, Morgane Gourlaouen^3^, Angélique Angot^3^, Mamadou Niang^4^ , Isaac Dah^5^, Kouramoudou Berete^6^, Adama Diakite^7^, Fatou Tall Lo^8^, Clement Meseko^9^, Emilie Go-Maro^10^, Valeria D’Amico^1^, Viviana Valastro^1^, Baba Soumare^3^, Paola De Benedictis^1^, Isabella Monne^1^, Valentina Panzarin^1^**

1 Istituto Zooprofilattico Sperimentale delle Venezie, Legnaro, Italy

2 Department of Public Health, University of Pavia, Pavia, Italy

3 Food and Agriculture Organization of the United Nations, Rome, Italy

4 Food and Agriculture Organization of the United Nations, Accra, Ghana

5 Laboratoire National Vétérinaire de Yaoundé, Yaoundé, Cameroon

6 Laboratoire Central de Diagnostic Vétérinaire de Conakry, Conakry, Guinea

7 Laboratoire Central Vétérinaire de Bamako, Bamako, Mali

8 Laboratoire National d’Elevage et de Recherches Vétérinaires de Dakar, Dakar, Senegal

9 National Veterinary Research Institute, Vom, Nigeria

10 Laboratoire Central Vétérinaire de Lomé, Lomé, Togo

**S1 Table. List of AIV samples employed for DSe determination and results.** CS: cloacal swab; OPS: oropharyngeal swab; O: organs; Liq: liquid reagent; Lyo: Qscript lyo 1-step (Quantabio); Pos: positive; Neg: negative. Samples positive for the viral target and showing negative IC (exogenous internal control) are considered conforming.

| **Sample ID** | **Region** | **Origin** | **Year** | **Subtype** | **Clade** | **Matrix** | **AIV#1** | | | | **H5#1** | | **H5#2** | |
| --- | --- | --- | --- | --- | --- | --- | --- | --- | --- | --- | --- | --- | --- | --- |
|  |  |  |  |  |  |  | Liq | | Lyo | | Liq | Lyo | Liq | Lyo |
|  |  |  |  |  |  |  | M gene | IC | M gene | IC |  |  |  |  |
| 17RS882-5 | Africa | duck | 2017 | H5N8 | 2.3.4.4b | CS | 19.5 | 26.3 | 20.1 | 24.1 | 23.5 | 23.6 | Pos | Pos |
| 17RS882-29 | Africa | duck | 2017 | H5N8 | 2.3.4.4b | OPS | 20.6 | 23.6 | 20.8 | 23.2 | 22.3 | 23.5 | Pos | Pos |
| 17RS882-40 | Africa | duck | 2017 | H5N8 | 2.3.4.4b | OPS | 22.4 | 23.9 | 22.9 | 23.4 | 25.9 | 26.3 | Pos | Pos |
| 17RS167-19 | Africa | chicken | 2017 | H5N8 | 2.3.4.4b | OPS | 21.1 | 23.6 | 21.5 | 23.4 | 23.4 | 24.0 | Pos | Pos |
| 17RS167-22 | Africa | goose | 2017 | H5N8 | 2.3.4.4b | CS | 25.7 | 23.8 | 25.9 | 23.3 | 27.2 | 28.6 | Pos | Pos |
| 17RS654-01 | Asia | chicken | 2016 | H5N8 | 2.3.4.4b | O | 20.1 | 23.4 | 20.2 | 22.9 | 22.5 | 23.1 | Pos | Pos |
| 17RS654-03 | Asia | chicken | 2016 | H5N8 | 2.3.4.4b | OPS | 22.7 | 24.7 | 23.2 | 24.2 | 25.6 | 26.0 | Pos | Pos |
| 17RS654-08 | Asia | chicken | 2017 | H5N8 | 2.3.4.4b | O | 25.8 | 25.3 | 26.3 | 24.6 | 28.7 | 29.9 | Pos | Pos |
| 17RS115-4 | Africa | white winged/black tern | 2017 | H5N8 | 2.3.4.4b | O | 33.0 | 25.4 | Neg | 24.0 | 36.2 | Neg | Neg | Neg |
| 17RS115-9 | Africa | duck | 2017 | H5N8 | 2.3.4.4b | O | 20.3 | 25.9 | 20.1 | 24.1 | 24.4 | 23.8 | Pos | Pos |
| 17RS115-10 | Africa | duck | 2017 | H5N8 | 2.3.4.4b | O | 24.0 | 24.7 | 24.1 | 23.8 | 27.3 | 28.6 | Pos | Pos |
| 17RS88-14 | Asia | pheasant | 2016 | H5N8 | 2.3.4.4b | O | 30.6 | 25.0 | 30.7 | 24.0 | 33.5 | 38.2 | Pos | Pos |
| 17RS804-76 | Africa | chicken | 2016 | H5N1 | 2.3.2.1c | O | 26.0 | 25.6 | 25.8 | 24.5 | 27.3 | 28.7 | Pos | Pos |
| 17RS804-82 | Africa | chicken | 2016 | H5N1 | 2.3.2.1c | O | 26.9 | 25.2 | 26.6 | 24.1 | 28.3 | 29.8 | Pos | Pos |
| 16VIR4304-1 | Africa | chicken | 2016 | H5N1 | 2.3.2.1c | O | 20.4 | Neg | 20.6 | Neg | 23.4 | 23.3 | Pos | Pos |
| 16VIR4304-5 | Africa | chicken | 2016 | H5N1 | 2.3.2.1c | O | 18.6 | Neg | 21.3 | Neg | 25.2 | 25.7 | Pos | Pos |
| 16VIR4304-9 | Africa | chicken | 2016 | H5N1 | 2.3.2.1c | O | 17.4 | 16.0 | 17.3 | Neg | 20.3 | 20.8 | Pos | Pos |
| 16VIR4304-13 | Africa | chicken | 2017 | H5N1 | 2.3.2.1c | O | 17.6 | Neg | 17.1 | Neg | 22.4 | 20.8 | Pos | Pos |
| 16VIR4304-21 | Africa | duck | 2016 | H5N1 | 2.3.2.1c | O | 20.8 | 26.9 | 20.6 | 24.5 | 23.8 | 23.8 | Pos | Pos |
| 16VIR3791-12 | Africa | chicken | 2016 | H5N1 | 2.3.2.1c | O | 18.3 | Neg | 18.0 | 24.1 | 27.2 | 23.4 | Pos | Pos |
| 16VIR3791-21 | Africa | duck | 2016 | H5N1 | 2.3.2.1c | O | 24.0 | 24.9 | 23.2 | 23.8 | 30.1 | 28.1 | Pos | Pos |
| VRD-15-KN76_18RS1971-2 | Africa | avian | 2015 | H5N1 | 2.3.2.1c | O | 23.6 | 26.5 | 23.6 | 25.9 | 31.6 | 29.7 | Pos | Pos |
| VRD-16-BA110_18RS1971-12 | Africa | avian | 2016 | H5N1 | 2.3.2.1c | O | 21.6 | 25.9 | 21.2 | 24.4 | 28.4 | 26.0 | Pos | Pos |
| VRD-16-KB210_18RS1971-14 | Africa | avian | 2016 | H5N1 | 2.3.2.1c | O | 26.7 | Neg | 23.0 | Neg | Neg | 27.5 | Pos | Pos |

**S2 table. List of RABV samples employed for DSe determination and results.** Liq: liquid reagent; Lyo: Qscript lyo 1-step (Quantabio); Pos: positive; Neg: negative. * Samples positive for the viral target and resulting negative in the β‑actin assay (or with Cq values over the cut-off, i.e. 32 Cq) are considered conforming.

| **Sample ID** | **Country** | **Origin** | **Year** | **Lineage** | **β-Actin** | **RABV#1** | | **RABV#2** | |
| --- | --- | --- | --- | --- | --- | --- | --- | --- | --- |
|  |  |  |  |  | Liq | Liq | Lyo | Liq | Lyo |
| 19RD/1345 | Brazil | Cow | 2011 | American indigenous | 31.5 | 27.2 | 26.9 | Pos | Pos |
| 19RD/1363 | Brazil | Cow | 2011 | American indigenous | 31.7 | 27.0 | 26.2 | Pos | Pos |
| 11RS/3570 | Italy (ex-India) | Human | 2011 | Arctic-like-1 | 25.9 | 25.1 | 25.0 | Pos | Pos |
| 11VIR/1039 | France | Fox | 1990 | Cosmopolitan (WE) | 33.1* | 21.4 | 21.3 | Pos | Pos |
| 15RD/772 | Greece | Fox | 2012 | Cosmopolitan (EE) | 31.9 | 22.1 | 22.0 | Pos | Pos |
| 07VIR/6097 | Niger | Dog | 2007 | Africa 2 | 25.4 | 21.4 | 21.0 | Pos | Pos |
| 12RS/639 | Spain (ex-Morocco) | Dog | 2010 | Cosmopolitan (AF 1) | 28.1 | 23.3 | 22.6 | Pos | Pos |
| 10VIR/5493 | Botswana | Dog | 2009 | Cosmopolitan (AF 1) | 23.7 | 21.4 | 20.9 | Pos | Pos |
| 10VIR/5493 | Botswana | Honey Badger | 2009 | Africa 3 | 24.6 | 22.5 | 22.1 | Pos | Pos |
| 07VIR/6235-1 | Mauritania | Dog | 2005 | Africa 2 | Neg* | 23.8 | 23.7 | Pos | Pos |
| 07VIR/6235-8 | Mauritania | Dog | 2006 | Africa 2 | 29.3 | 22.8 | 22.7 | Pos | Pos |
| 20RD/308-2 | Benin | Dog | 2018 | Africa 2 | 24.1 | 23.9 | 23.5 | Pos | Pos |
| 20RD/308-3 | Benin | Dog | 2018 | Africa 2 | 25.4 | 22.7 | 22.3 | Pos | Pos |
| 20RD/308-4 | Benin | Dog | 2018 | Africa 2 | 27.3 | 25.3 | 25.0 | Pos | Pos |
| 20RD/308-6 | Benin | Dog | 2018 | Africa 2 | 27.1 | 30.3 | 29.7 | Pos | Pos |
| 18RD/1557-2 | Guinea | Dog | 2018 | Africa 2 | 18.9 | 15.9 | 16.0 | Pos | Pos |
| 19RD/1538-5 | Italy (ex-Zanzibar) | Human | 2019 | Cosmopolitan (AF 1) | 18.1 | 15.8 | 15.8 | Pos | Pos |
| 12RS/978-8 | Brazil | Cow | 2010 | American indigenous | 18.4 | 17.1 | 17.7 | Pos | Pos |
| 11VIR/673-11 | Italy | Fox | 2011 | Cosmopolitan (WE) | 22.3 | 17.0 | 17.1 | Pos | Pos |
| 21RD/243-2 | Mali | Dog | 2017 | Africa 2 | 30.5 | 30.7 | 30.7 | Pos | Pos |
| 21RD/243-4 | Mali | Dog | 2019 | Africa 2 | 31.4 | 22.0 | 22.3 | Pos | Pos |
| 21RD/243-5 | Mali | Dog | 2020 | Africa 2 | 37.5* | 25.0 | 25.6 | Pos | Pos |
| 21RD/243-7 | Mali | Dog | 2020 | Africa 2 | 32.7* | 22.9 | 23.8 | Pos | Pos |
| 21RD/243-10 | Mali | Dog | 2020 | Africa 2 | 33.3* | 24.4 | 25.0 | Pos | Pos |
| 21RD/243-15 | Mali | Dog | 2020 | Africa 2 | 34.0* | 28.0 | 28.0 | Pos | Pos |
| 21RD/243-16 | Mali | Dog | 2020 | Africa 2 | 34.4* | 28.9 | 29.1 | Pos | Pos |

**S3 table. Composition of the AIV panel used for the inter-laboratory reproducibility exercise, and expected results.** Pos: positive; Neg: negative. * According to the unified nomenclature for HPAI A (H5) used by the World Health Organization (WHO) Global Influenza Surveillance and Report System (GISRS); ** According to the updated Newcastle disease viruses nomenclature (Dimitrov et al., 2019; doi:10.1016/j.meegid.2019.103917)

| **Code** | **Virus** | **Year** | **Type** | **Clade/**  **Lineage** | **Pathotype** | **Virus strain** | **Expected results** | | |
| --- | --- | --- | --- | --- | --- | --- | --- | --- | --- |
|  |  |  |  |  |  |  | **AIV#2** | **H5#1** | **H5#2** |
| L01 | AIV | 2019 | H5N8 | 2.3.4.4B* | HPAI | 19VIR8424-20 | Pos | Pos | Pos |
| L02 | NDV | 2019 | APMV-1 | VII.1.1** | Virulent | 20VIR6516 | Neg | Neg | Neg |
| L03 | AIV | 2020 | H5N8 | 2.3.4.4B* | HPAI | 14_20VIR205-19 | Pos | Pos | Pos |
| L04 | AIV | 2019 | H5N6 | 2.3.4.4B* | HPAI | 19VIR8424-2 | Pos | Pos | Pos |
| L05 | AIV | 2013 | H7N7 |  | HPAI | 13VIR4527-11 | Pos | Neg | Neg |
| L06 | AIV | 2018 | H7N7 |  | LPAI | 18VIR4932-2 | Pos | Neg | Neg |
| L07 | AIV | 2019 | H9N2 | G1 | LPAI | 19VIR8424-15 | Pos | Neg | Neg |
| L08 | AIV | 2020 | H5N5 | 2.3.4.4B* | HPAI | 13776-1_20VIR7282-13 | Pos | Pos | Pos |
| L09 | AIV | 2020 | H5N1 | 2.3.4.4B* | HPAI | 20VIR7301-206 | Pos | Pos | Pos |
| L10 | NDV | 2020 | APMV-1 | XXI.2** | Virulent | 20VIR3543-9 | Neg | Neg | Neg |
| L11 |  |  |  |  |  | Negative allantoic fluid | Neg | Neg | Neg |
| L12 |  |  |  |  |  | Negative allantoic fluid | Neg | Neg | Neg |

**S4 table. Composition of the RABV panel used for inter-laboratory reproducibility exercise and expected results.** Pos: positive; Neg: negative; WP: weakly positive.

| **Code** | **Sample and virus strain** | **Expected results** | | |
| --- | --- | --- | --- | --- |
|  |  | **β-actin** | **RABV#1** | **RABV#2** |
| Rabies01 | Negative | Neg | Neg | Neg |
| Rabies02 | Negative for RABV, with not infected CNS | Pos | Neg | Neg |
| Rabies03 | RABV Africa 2, with CNS | Pos | Pos | Pos |
| Rabies04 | RABV CVS-11, with CNS | WP | Pos | Pos |
| Rabies05 | RABV Africa 3, with CNS | Pos | Pos | Pos |
| Rabies06 | Negative for RABV, with not infected CNS | Pos | Neg | Neg |
| Rabies07 | RABV Cosmopolitan (AF1), with CNS | Pos | Pos | Pos |
| Rabies08 | RABV CVS-11, with CNS | WP | Pos | Pos |
| Rabies09 | RABV Africa 2, with CNS | Pos | Pos | Pos |
| Rabies10 | RABV Africa 3, with CNS | Pos | Pos | Pos |
| Rabies11 | Negative | Neg | Neg | Neg |
| Rabies12 | RABV Cosmopolitan (AF1), with CNS | Pos | Pos | Pos |
| Rabies13 | Negative for RABV, with not infected CNS | Pos | Neg | Neg |

**S5 table. Inventory of the freeze-dried RT-PCR kits marketed at the time of the study.** RT: room temperature

| **Name** | **Manufacturer** | **Recommended storage conditions** | **Format of the smallest kit size** | **Smallest volume to be re-hydrated** | **Storage after re-hydratation** |
| --- | --- | --- | --- | --- | --- |
| Qscript lyo 1-step | Quantabio, MA, USA | 4°C or RT,  9 months | 3× 8-tube strips | Single tube (1 rnx) | Not applicable |
| Takyon Dry One-Step RT Probe MasterMix No Rox | Eurogentec, Belgium | 15-35°C,  18 months | 3× bottles for 50 rnx each | 50 rnx | 4°C, 24 hours  or  -20°C, 1 month |
| 5X CAPITAL qRT PCR Probe Master Mix lyophilized | Biotech rabbit, Germany | RT or below, 24 months | 2× tubes for 100 rnx each | 100 rnx | -20°C,  12 months |
| SCRIPT RT-qPCR ProbesMaster Lyophilisate | Jena Bioscience, Germany | RT,  6 months in sealed package | 24× 8-tube strips | Single tube  (1 rnx) | Not applicable |
| Lyophilized One Step qRT PCR | Bio-Techne SRL, Italy | RT,  18 months | 3× glass ampules for 50 rnx each | 50 rnx | -20°C,  6 months |
| ViPrimePLUS Lyophilized One Step qRT-PCR Master Mix | Vivantis, Malaysia | RT,  18 months | 3× tubes for 50 rnx each | 50 rnx | -20°C,  18 months |

**S6 table. LoD obtained for RABV#1 and RABV#3 assays using the liquid reagent and the Qscript lyo 1-step.** The RABV#1 and RABV#3 assays were employed for the evaluation. Liq: liquid reagent; Lyo: Qscript lyo 1-step (Quantabio). Each dilution is expressed as RNA copies/µl and was tested in triplicate. Cq values are reported as the average of the three replicates with standard deviation. Neg: negative; LoD is highlighted in grey; n.p.: not performed.

|  | **RNA copies/µl** |  | |  | |  | |
| --- | --- | --- | --- | --- | --- | --- | --- |
| **ASSAY** |  | **DUVV** | | **MOKV** | | **IKOV** | |
|  |  | Liq | Lyo | Liq | Lyo | Liq | Lyo |
|  | 10^5^ | 25.0 ± 0.1 | n.p. | 24.0 ± 0.2 | n.p. | 34.0 ± 0.1 | 35.2 ± 0.2 |
|  | 10^4^ | 27.8 ± 0.3 | 27.4 ± 0.1 | 27.1 ± 0.6 | 27.4 ± 0.3 | Neg | Neg |
|  | 10^3^ | 31.1 ± 0.3 | 30.9 ± 0.1 | 30.3 ± 0.2 | 30.6 ± 0.2 | Neg | Neg |
| RABV#1 | 10^2^ | 35.0 ± 0.5 | Neg | 33.8 ± 0.3 | 34.2 ± 0.3 | Neg | Neg |
|  | 10 | Neg | Neg | 36.3 ± 0.8 | Neg | Neg | Neg |
|  | 1 | Neg | Neg | Neg | Neg | Neg | Neg |
|  | 10^5^ | 21.8 ± 0.2 | 21.8 ± 0.3 | 22.4 ± 0.5 | 22.3 ± 0.3 | 21.8 ± 0.1 | 21.3 ± 0.1 |
|  | 10^4^ | 25.2 ± 0.2 | 25.2 ± 0.3 | 26.0 ± 0.3 | 25.7 ± 0.1 | 24.9 ± 0.3 | 24.7 ± 0.1 |
| RABV#3 | 10^3^ | 28.1 ± 0.9 | 28.1 ± 0.7 | 28.3 ± 0.2 | 28.5 ± 0.1 | 27.9 ± 0.0 | 27.9 ± 0.4 |
|  | 10^2^ | 31.7 ± 0.6 | 31.7 ± 0.2 | 32.0 ± 0.9 | 31.3 ± 0.6 | 32.2 ± 1.49 | 31.4 ± 0.2 |
|  | 10 | 35.3 ± 0.9 | 35.3 ± 0.2 | Neg | 35.6 ± 0.6 | Neg | 35.0 ± 0.3 |
|  | 1 | Neg | Neg | Neg | Neg | Neg | Neg |

**S7 table**. **Qscript lyo 1-step (Quantabio) stability tests**. Cq values are reported as the mean of the three replicates with standard deviation. Liq: liquid reagent; Lyo: Qscript lyo 1-step (Quantabio).

| Storage conditions | H5N1 dilutions | AIV#1 | | H5#1 | |
| --- | --- | --- | --- | --- | --- |
|  |  | **Liq** | **Lyo** | **Liq** | **Lyo** |
| 4°C (i.e. optimal storage condition)  for 2 months | 10^4.5^ | 20.1 ± 0.1 | 21.2 ± 0.1 | 20.4 ± 0.0 | 19.9 ± 0.2 |
|  | 10^1.5^ | 30.4 ± 0.1 | 31.5 ± 0.1 | 30.5 ± 0.5 | 30.3 ± 0.0 |
|  | 10^0.5^ | 34.2 ± 0.3 | 35.3 ± 0.7 | 33.6 ± 0.8 | 34.0 ± 0.4 |
| Room temperature  for 9 months | 10^4.5^ | 18.1 ± 0.3 | 19.3 ± 0.3 | 19.5 ± 0.3 | 21.2 ± 0.0 |
|  | 10^1.5^ | 26.0 ± 0.2 | 27.6 ± 0.2 | 27.8 ± 0.3 | 30.1 ± 0.2 |
|  | 10^0.5^ | 28.5 ± 0.2 | 30.3 ± 0.3 | 30.1 ± 0.4 | 33.4 ± 2.7 |
| 30°C  for 10 days | 10^4.5^ | 20.2 ± 0.1 | 20.2 ± 0.1 | 22.2 ± 0.1 | 22.5 ± 0.2 |
|  | 10^1.5^ | 30.1 ± 0.2 | 30.5 ± 0.2 | 32.5 ± 0.2 | 34.5 ± 0.7 |
|  | 10^0.5^ | 33.7 ± 0.4 | 34.1 ± 0.8 | 36.0 ± 0.7 | 38.4 ± 1.3 |
